# Fructose plus High-Salt Diet in Early Life Results in Salt-Sensitive Cardiovascular Changes in Mature Male Sprague Dawley Rats

**DOI:** 10.3390/nu13093129

**Published:** 2021-09-08

**Authors:** Peter E. Levanovich, Charles S. Chung, Dragana Komnenov, Noreen F. Rossi

**Affiliations:** 1 Department of Physiology, Wayne State University, Detroit, MI 48201, USA; fy7541@med.wayne.edu (P.E.L.); cchung@wayne.edu (C.S.C.); 2 Department of Internal Medicine, Wayne State University, Detroit, MI 48201, USA; fv6083@wayne.edu; 3 John D. Dingell VA Medical Center, Detroit, MI 48201, USA

**Keywords:** aortic stiffness, fructose, glucose, hypertension, left ventricular diastolic dysfunction, pulse wave velocity, renal resistive index

## Abstract

Fructose and salt intake remain high, particularly in adolescents and young adults. The present studies were designed to evaluate the impact of high fructose and/or salt during pre- and early adolescence on salt sensitivity, blood pressure, arterial compliance, and left ventricular (LV) function in maturity. Male 5-week-old Sprague Dawley rats were studied over three 3-week phases (Phases I, II, and III). Two reference groups received either 20% glucose + 0.4% NaCl (GCS-GCS) or 20% fructose + 4% NaCl (FHS-FHS) throughout this study. The two test groups ingested fructose + 0.4% NaCl (FCS) or FHS during Phase I, then GCS in Phase II, and were then challenged with 20% glucose + 4% NaCl (GHS) in Phase III: FCS-GHS and FHS-GHS, respectively. Compared with GCS-GCS, systolic and mean pressures were significantly higher at the end of Phase III in all groups fed fructose during Phase I. Aortic pulse wave velocity (PWV) was elevated at the end of Phase I in FHS-GHS and FHS-FHS (vs. GCS-GCS). At the end of Phase III, PWV and renal resistive index were higher in FHS-GHS and FHS-FHS vs. GCS-GCS. Diastolic, but not systolic, LV function was impaired in the FHS-GHS and FHS-FHS but not FCS-FHS rats. Consumption of 20% fructose by male rats during adolescence results in salt-sensitive hypertension in maturity. When ingested with a high-salt diet during this early plastic phase, dietary fructose also predisposes to vascular stiffening and LV diastolic dysfunction in later life.

## 1. Introduction

The prevalence of hypertension has been increasing in recent decades in the United States both independently and concurrently with diabetes [1]. Elevated fructose consumption has been implicated in metabolic disorders and subsequent cardiovascular morbidity [2,3,4]. In pre-clinical models, high levels of fructose consumption—often exceeding 60% of daily caloric intake—elicit hypertension and cardiovascular dysfunction, and implicate insulin signaling as the pathogenic mechanism [4,5]. Ingestion of 20% fructose in drinking water together with high-salt chow, which is more representative of the diet ingested by the upper quintile in humans in the United States, results in sodium and fluid retention in rats, enhanced sympathetic activation, and inadequate suppression of plasma renin activity, leading to a hypertensive state prior to development of frank metabolic syndrome or diabetes mellitus [6,7].

Adolescence is marked by the continuous development and growth of physiologic systems. In early stages of life, various systems undergo substantial ontogenetic changes, some of which are susceptible to modulation by external stimuli. Several studies have demonstrated the effect of excess fructose consumption on cardiovascular systems in adults [8,9,10]. However, little is understood regarding the impact of fructose-rich diets during adolescence on cardiovascular parameters later in life [11,12,13,14,15]. The major consumers of fructose are adolescents and young adults, with sugar-sweetened beverages representing the main source. Fructose intake in adolescents accounts for nearly 20% of daily energy consumption [16,17].

Western diets use high-fructose corn syrup extensively as a sweetener but are also high in sodium content [3]. Since pre-clinical studies indicate that a diet high in both fructose and salt results in hypertension [5,6,10,11], aortic stiffness, and early diastolic dysfunction [12], the question arises whether ingestion of high fructose and salt during a critical period early in life predisposes to salt-sensitive hypertension and cardiovascular dysfunction in later life. This window of plasticity during adolescence has been well recognized in behavioral science [18,19]. Likewise, with cardiovascular development, rat models have shown that interventions during critical time periods of ontogeny may modulate susceptibility to hypertension later in life. Insights into post-gestational influences on arterial pressure have been garnered predominantly from studies using genetically hypertensive-strain rats such as Dahl salt-sensitive and spontaneously hypertensive rats to investigate the impact on disease progression [20]. For example, four-week treatment of young spontaneously hypertensive rats with angiotensin-converting enzyme inhibition attenuated development of elevated blood pressure in later life [21]. The converse has not been given much attention, namely, whether factors such as diet or environment during this critical developmental period may adversely alter cardiovascular parameters in maturity, even in a rat strain that is not genetically prone to hypertension.

One in five adolescents in the United States are now considered pre-diabetic [22]. This increasing incidence of pre-diabetes raises the potential of cardiovascular dysfunction later in life that can be further impacted by poor dietary habits at this stage. Thus, the purpose of the present study was to investigate whether exposure to high fructose with or without high salt during the critical adolescent period will lead to hypertension and cardiovascular dysfunction in response to high-salt diet later in life. We hypothesized that rats consuming 20% fructose plus with 4% sodium diet during five to eight weeks of age (comparable to human pre- and early adolescence) [19,23] will develop elevated salt-sensitive blood pressure, reduced arterial compliance, and left ventricular diastolic dysfunction in adulthood when challenged with high dietary sodium in the absence of fructose.

## 2. Materials and Methods

All animal procedures and protocols were approved by the Wayne State University Institutional Animal Care and Use Committee (Protocol #19-03-1001). Animal care and experimentation was conducted in accordance with the guidelines and principles articulated in the National Institutes of Health Guide for the Care and Use of Laboratory Animals. Male Sprague Dawley rats (Envigo Sprague Dawley, Shelby, MI, USA) were housed under controlled conditions (21–23 °C; 12 h light and 12 h dark cycles, lighting period beginning at 6 a.m.).

### 2.1. Dietary Regimen

**Figure 1:**
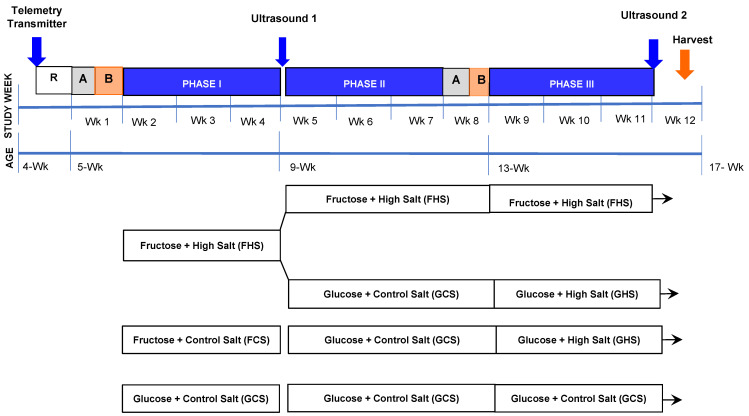
**Schematic of the Timeline of Experimental Protocols and Study Phases.** Rat age and study week are depicted across the timeline. R, recovery period; A, acclimation to metabolic cages; B, baseline. Surgery for telemetry transmitter placement and ultrasound studies are as indicated. Groups are subsequently depicted by their sugar-salt intake in Phases I and III.

Upon arrival, rats were permitted to acclimate for at least 48 h and provided standard lab chow and water, ad libitum. As depicted in Figure 1, when rats reached ~4.5 weeks of age, a hemodynamic transmitter was implanted (as described in surgical procedures) and the animal was permitted to recover in individual standard polyurethane caging. One week later, rats were placed into metabolic housing units (Tecniplast USA, West Chester, PA, USA) and provided milled chow containing either 20% glucose and 0.4% Na^+^ (glucose control salt, GCS; ModTest Diet^®^ 5755-5WZZ; St. Louis, MO, USA) or 20% fructose and 0.4% Na^+^ (fructose control salt, FCS; ModTest Diet^®^ 5755-5W3Y; St. Louis, MO, USA). Rats were permitted a 3 day acclimation period followed by a 3 day baseline period where food and water were provided ad libitum and baseline hemodynamic data were recorded by telemetry. Then, the rats entered Phase I (Figure 1; study weeks 2 to 4, inclusive): GCS rats (*n* = 9) continued on the same diet. Rats receiving FCS chow were then randomly assigned to continue FCS (*n* = 9) or placed on 20% fructose and 4.0% Na^+^ (fructose high salt, FHS; ModTest Diet^®^ 5755-5WZ8; *n* = 18; St. Louis, MO, USA) for three weeks. At this time, a pair feeding paradigm was initiated to achieve equal caloric intake among the groups on a day-to-day basis. Water continued to be provided ad libitum. Food and water intake and urine output were assessed daily. In Phase II (Figure 1, study weeks 5 to 7, inclusive), all rats were returned to standard individual shoebox housing units. Rats on GCS feed were maintained on this diet for the remainder of this study, including Phase III. Rats on FCS feed were then placed on GCS chow. The rats on FHS chow were then further randomly assigned to receive either GCS feed (*n* = 9) or to continue the FHS diet (*n* = 9). The rats on FHS chow during Phase II remained on FHS through to the end of this study.

After 3 weeks, the rats were again placed into metabolic cages and permitted to acclimate to the change in caging for three days prior to initiating Phase III (Figure 1; study weeks 9 to 11, inclusive). FCS- and FHS-fed rats that had been shifted to a GCS feed in Phase II were then subjected to a high-salt challenge without fructose for the remainder of the protocol using a 20% glucose and 4.0% Na^+^ chow (glucose high salt, GHS; ModTest Diet^®^ 5755-5WOW). This produced four groups characterized by their dietary regimens in the early and late phases—Phase 1 and Phase III, respectively (Figure 1). The groups are named based on their diets during Phases I and III: (a) GCS-GCS, (b) FCS-GHS, (c) FHS-GHS, and (d) FHS-FHS. Rats were maintained on these diets for an additional three weeks; thereafter, terminal studies were performed.

### 2.2. Ultrasonography

At the end of Phases I and III, rats were anesthetized in an induction chamber using 3% isoflurane and transferred to a pre-heated electronic ECG platform where 1–1.5% isoflurane was delivered via nosecone to maintain a sufficient plane of anesthesia. Fur from the chest and abdominal area was removed using an electric shaver followed by application of depilatory cream (Church & Dwight Co., Inc., Erwing, NJ, USA). Electrode gel was placed on each of the ECG strips where the rat’s limbs were held in place using tape. Body temperature was measured via a rectal probe and contact gel preheated to 37 °C was applied before performing echocardiography according to standard methods [24,25].

Image acquisition was conducted using the Vevo3100 Imaging system and MX250S transducer (Fujifilm Visualsonics, Inc., Toronto, ON, Canada). Assessment of left ventricular (LV) dimensions and systolic function was performed using a short axis view in M-mode at the level of the papillary muscle. Left ventricular (LV) diastolic filling and function were assessed using pulsed wave Doppler of transmitral blood flow velocities. These were located using color imaging superimposed over an apical four-chamber view. Further assessment of LV diastolic function was conducted using tissue Doppler imaging (TDI) near the mitral annulus measured along the apical axis. Pulse wave velocity (PWV) determination within the aortic arch was made via the determination of pulse transit time from the aortic root to a point within the aortic arch. Distance between these points was measured using a B-mode image of this anatomical segment. Aortic PWV was calculated as the difference in pulse transit time (calculated using the ECG tracing as a reference) measured at these two points divided by the distance between them.

Renal resistive index (RRI) was determined using pulsed Doppler measurements along the left main renal artery. RRI was calculated by taking the difference between systolic and diastolic velocity divided by the diastolic velocity during each respective cardiac cycle [26]. Data analysis was performed offline using VevoLab and VevoVasc software (Fujifilm Visualsonics, Inc., Toronto, ON, Cananda) in blinded fashion.

### 2.3. Surgical Procedures

All surgical procedures were conducted under intraperitoneal ketamine (80 mg/kg; Mylan Institutional, LLC Rockford, IL, USA) and xylazine (10 mg/kg; Akorn Animal Health, Inc., Lake Forest, IL, USA) anesthesia and subcutaneous administration of buprenorphine SR (0.3 mg/kg) for analgesia.

Hemodynamic Transmitter Placement: Following right femoral artery isolation, a small arterial incision was made and the gel-filled catheter of the hemodynamic transmitter (HDS-10, Data Sciences International, New Brighton, MN, USA) was inserted into the vessel and advanced into the abdominal aorta. The catheter was then anchored in place to the femoral artery using 3-0 silk suture (Ethicon, Johnson & Johnson, New Brunswick, NJ, USA) and the transmitter body was subcutaneously tunneled to the right flank. Subcutaneous adipose tissue was reapproximated around the surgical sight and the incision was closed using surgical staples.

Vascular Catheter Placement: At the end of Phase III and after the second ultrasonography study, catheters were placed into the left carotid artery and external jugular vein using ketamine and xylazine as above, and previously performed in our laboratory [7,27]. Catheters were secured with 3-0 silk suture and tunneled subcutaneously to the base of the neck and exteriorized. All incisions were closed using 4-0 prolene suture (Ethicon, Johnson & Johnson, New Brunswick, NJ, USA). The catheters were then filled with heparinized saline (1000 units/mL). The rats were then permitted to recover in individually housed polyurethane cages.

### 2.4. Analytical Measurements and Calculations

Hemodynamic Telemetry: Acquisition of hemodynamic data was conducted using Ponemah software (Data Sciences International, New Brighton, MN, USA). Systolic blood pressure (SBP), diastolic blood pressure (DBP), mean arterial pressure (MAP), and heart rate (HR) were sampled for 10 s every 4 min at a sampling rate of 500 samples/second. Pulse pressure (PP) was calculated separately using these values. Baseline measurements were averaged over three days after cage acclimation. Sampling was performed at this rate continuously throughout Phases I and III within the metabolic cages.

Metabolic and Hormonal Assessment: During Phases I and III, daily chow consumption was measured gravimetrically and caloric and sodium (Na^+^) intake values were determined based on dietary profiles for each feed.

At the end of Phase III and prior to terminal harvest, food was removed from caging at the beginning of the morning light cycles and terminal procedures were conducted at a minimum of 6 h later to promote a semi-fasting state (as permitted by our Animal Use Committee). Glucose levels were determined using a One-Touch Ultra glucose monitor (LifeScan, Inc., Malpitas, CA, USA) on 50 µL blood from the arterial catheter. Arterial blood was collected into pre-chilled tubes containing sodium ethylenediaminetetraacetic acid (EDTA) for plasma renin activity (PRA) and into separate pre-chilled tubes containing 120 μL of 500 mM sodium EDTA, 125 mM phenanthroline, 1 mM phenylmethanesulfonyl fluoride, 20 mM pepstatin, 1 mM enalapril and 10× phosphatase inhibitor cocktail for insulin determinations. Once collected, blood was immediately centrifuged at 3000 rpm for 4 min at 4 °C. Plasma was stored at −70 °C until assay. PRA and insulin levels were measured using a standard Elisa kits (IBL International, Hamburg, Germany and Bertin Pharma SAS, Montingny-le-Bretonneux, France, respectively) Insulin sensitivity was calculated using the ratio of plasma glucose to insulin levels.

Rats were euthanized using sodium pentobarbital (120 mg/kg, IV) and hearts were excised and preserved in formalin solution for 24–48 h before embedding for histological assessment. Samples were stained with Mason’s Trichrome dye and images were acquired at 40× magnification (Leica CTR5000, Leica Microsystems Inc., Buffalo Grove, IL, USA).

Statistics: All values are presented as mean ± standard error (SE). One-way analysis of variance (ANOVA) was used to determine differences among groups with a Sidak’s multiple comparisons test for post hoc analysis. Two-way ANOVAs with repeated measures were performed to compare differences over time using a Sidak’s multiple comparisons test for post hoc analysis. A *p*-value less than 0.05 was considered statistically significant. Due to the technical nature of many of these experimental techniques and acquisition of data over an extended period, some data were not able to be acquired at each time point for each animal. When *n*-values deviate from the original assignment, the reason for missingness is provided and values imputed as the mean. Consistent with the use of a repeated measures design, animals with missing data in any phase were omitted from two-way ANOVA tests for the analysis of change over time.

## 3. Results

### 3.1. Metabolic and Humoral Profiles

Metabolic parameters are shown in Table 1. Initial and final rat weights did not differ among the groups on different dietary regimens. Plasma glucose and insulin levels did not vary among the groups and were comparable to either vendor specifications for standard Sprague Dawley rats on standard diets and rats fed control diets with no added sugar in similar studies [28,29,30]. The glucose:insulin ratio was significantly lower in the FHS-GHS and FHS-FHS groups.

Compared with the FCS-GHS group. Although the glucose:insulin ratio was nearly two-fold higher in the GCS-GCS rats compared with FHS-GHS and FHS-FHS rats, statistical significance was achieved only for the FHS-GHS (*p* < 0.01) but not for FHS-FHS groups (*p* = 0.0512). PRA was significantly reduced in FCS-GHS groups following high-salt challenge at the end of Phase III. FHS-GHS and FHS-FHS rats displayed a blunted inhibition of renin secretion.

Statistical differences among caloric intakes were observed following dietary changes from control salt to high-salt chow in Phase I, week 1 and in Phase III weeks 1 and 2 Caloric intakes were no different among the groups by of week 2 of Phase I and III except of the FCS-GHS group in Phase III that continued to ingest fewer calories (Table 2). Despite rigorous efforts to match intake among the groups, rats in the FHS-GHS and FHS-FHS diets had lower caloric intake only in week 1 of Phase I (and consequently lower calorie and sodium intakes compared with weeks 2 and 3 of Phase I, *p <* 0.05 vs. either calorie or sodium, respectively) after which their caloric intake was no different than that of the GCS-GCS group. High salt-fed rats consumed approximately 10-fold greater amount of sodium than that consumed by the control salt-fed rats, consistent with their respective dietary sodium diets.

### 3.2. Hemodynamic Effects

In Phase I, the addition of high salt in the diet of fructose-fed rats led to a progressive increase in mean arterial pressure that was significantly elevated after four days (data not shown). This increase was sustained for the subsequent two weeks. By the end of Phase I, the MAP increased in all groups consistent with expected changes in blood pressure with maturation; however, MAP was significantly higher in the rats receiving FHS diet during this phase (Table 3A). The FCS-GHS and FHS-GHS groups received GCS chow during Phase II and MAP at the beginning of Phase III did not differ from the MAP of the GCS-GCS group (data not shown). When FCS-GHS and FHS-GHS groups were placed on the GHS diet in Phase III, significant increases in MAP occurred within 1 week and these were sustained throughout the remainder of the phase. Final MAP pressure levels were comparable to those of FHS-FHS rats that had been on high-salt diet throughout all three phases (Table 3B). Systolic blood pressures at increased significantly in all groups on high-salt diet during Phase III compared with the GCS-GCS group that had ingested glucose and 0.4% salt chow throughout this study (*p* < 0.05). Diastolic blood pressures at the end of Phase I did not differ across any of the groups; therefore, the increases in MAP were driven predominately by systolic mechanisms. Pulse pressure, however, did not increase significantly. Additionally, no differences existed among groups when heart rate was assessed.

### 3.3. Measurements of Vascular Compliance

**Figure 2:**
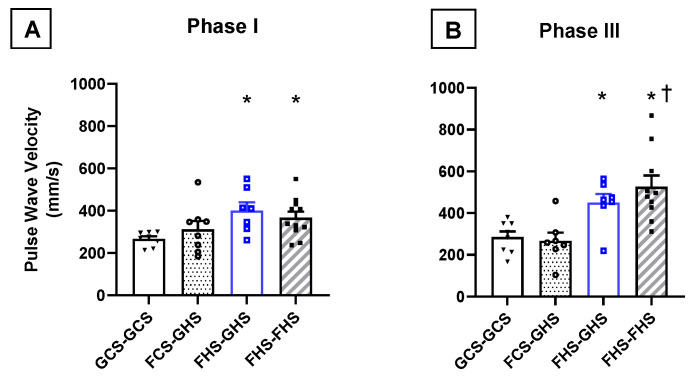
**Pulse Wave Velocity at the end of Phases I and III.** Pulse wave velocity (PWV) of the ascending aorta assessed (**A**) at the end of Phase I and (**B**) at the end of Phase III. Group labels are as described in the legend for Table 1. Values are the mean ± SE; *n* as depicted on the graphs. * *p* < 0.05 vs. GCS-GCS (by one-way ANOVA); ^†^ *p* < 0.05 vs. FHS-FHS in Phase I (by two-way ANOVA with repeated measures).

Echocardiography and ultrasonography studies were performed upon completion of Phases I and III. At the end of Phase I, FHS diet significantly increased PWV of the ascending thoracic aorta, thereby indicating decreased aortic compliance compared to the GCS-GCS group (Figure 2). By the end of Phase III, maintenance of the FHS diet throughout all three phases of the protocol led to a significantly greater PWV in the in FHS-FHS group compared with the GCS-GCS group that had glucose and control salt diet for the same duration. Notably, PWV was significantly higher in the FHS-FHS group at the end of Phase III compared with the same animals at the end of Phase I.

**Figure 3:**
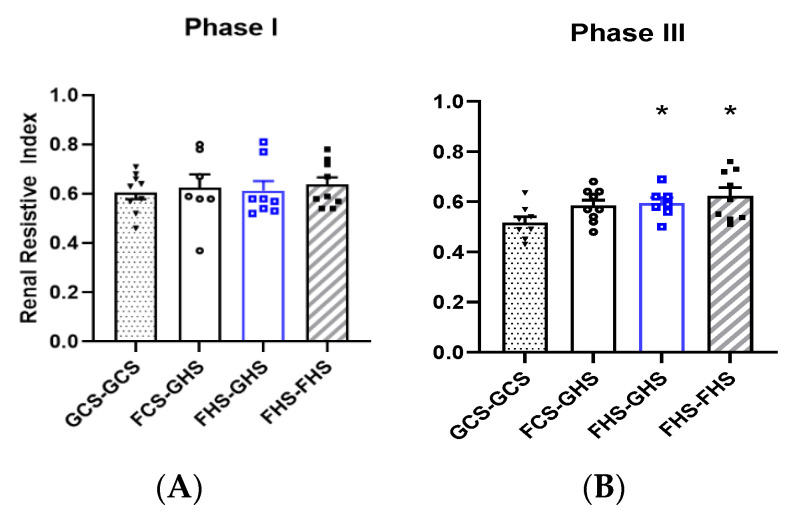
**Renal Resistive Index at the end of Phases I and III**. Renal resistive index (RRI) of the left main renal artery using doppler imaging (**A**) at the end of Phase I and (**B**) at the end of Phase III. Values are the mean ± SE; *n* as depicted on the graphs. * *p* < 0.05 vs. GCS-GCS in Phase III.

No differences were observed in the RRI among any of the groups at the end of Phase I (Figure 3). Similar to PWV values, FHS-GHS and FHS-FHS groups in Phase III displayed a statistically higher RRI than GCS-GCS rats. These trends were only elevated in Phase III and demonstrated no intra-group variability when compared to Phase I by two-way ANOVA. Note that due to anatomic and/or technical reasons, PWV and RRI were not able to be reliably assessed in each animal.

### 3.4. Echocardiographic Assessments

**Figure 4:**
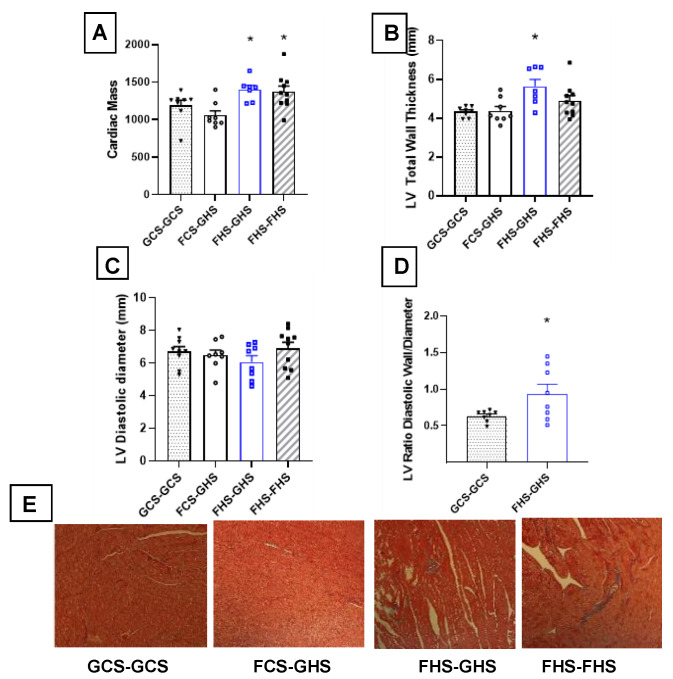
**Assessment of Left Ventricular Parameters in Phase III.** Images were acquired using a short axis view of the left ventricle (LV) via M-Mode. (**A**) Cardiac mass was the total wet weight of the heart after harvesting. (**B**) Total wall thickness was the sum of anterior and posterior LV wall widths. (**C**) LV diastolic diameter was the diameter of the LV at end diastole. (**D**) LV diastolic wall/diameter was calculated as the ratio of total wall thickness to the diameter of the LV at end-diastole. Values are the mean ± SE, *n* as indicated per group; * *p* < 0.05 vs. GCS-GCS. (**E**) Representative histological sections of LV tissue from each group. Group labels are as described in the legend for Table 1. Mason’s trichrome; 40× magnification.

Physical assessment of the LV was performed using a short axis view and M-mode imaging. Table 4 depicts the results of echocardiography of the LV performed at the end of Phase III. No significant differences of standard systolic function, such as ejection fraction and fractional shortening, were observed among the groups. LV mass was significantly greater in the FHS-GHS and FHS-FHS groups vs. the GCS-GCS group. Further assessment of structural morphology revealed significant increases in anterior and posterior wall thickness in the FHS-GHS group. These changes were only apparent during diastole. In the FHS-FHS group, LV thickness only reached significance in the anterior wall. When assessed as a ratio of total wall thickness (sum of anterior and posterior walls) to inner diameter of the LV during diastole, there was a significant reduction in the ratio in FHS-GHS rats when compared with that of the GCS-GCS group (Figure 4A–D). Figure 4E shows histological views typical of the LV myocardium from the four groups. Notably, collagen staining was observed in the tissues taken from the FHS-GHS and FHS-FHS rats. Taken together, these measurements are consistent with ventricular hypertrophy and concentric remodeling.

**Figure 5:**
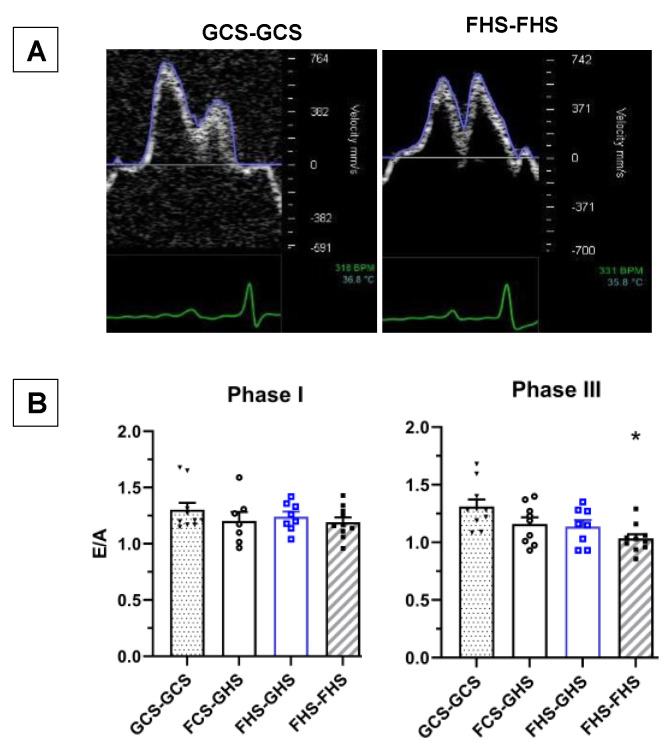
**Assessment of Diastolic Function.** (**A**) Representative Doppler images of transmitral flow patterns from GCS-GCS and FHS-FHS rats at the end of Phase III. (**B**) Ratio of early (E-wave) to late (A-wave) left ventricular filling (E-wave) left ventricular filling as index of diastolic function. Group labels are as described in the legend for Table 1. Values are the mean ± SE; *n* as indicated per group, * *p* < 0.05 vs. GCS-GCS.

Figure 5A shows representative Doppler images of transmitral flow from GCS-GCS and FHS-FHS rats at the end of Phase III. Table 5 shows values of diastolic function conducted by way of pulsed and tissue Doppler imaging at the end of Phase III. The ratio of early to late phase filling (E/A) demonstrated a significant reduction in the FHS-FHS group compared with the GCS-GCS group. The E/A ratios for each of the groups at the end of both Phase I and Phase III indicate that this parameter of diastolic dysfunction is significantly suppressed only after 12 weeks of the diet high in both fructose and salt (Figure 5B). Reductions in the E/A ratio in the FCS-GHS and FHS-GHS test groups were also observed, although these values did not achieve significance (*p* = 0.064 vs. GCS-GCS). Likewise, decreases in mitral valve deceleration time were also observed across all groups when compared to the GCS-GCS group. However, these were also only significant in the FHS-FHS group.

## 4. Discussion

The major findings of this study support the hypothesis that consumption of fructose plus high-salt diet during pre- and early adolescence results in measurable deleterious cardiovascular effects in adulthood when ingesting high dietary sodium without fructose. Specifically, ingestion of high fructose either alone or with high salt during this early critical period of life resulted in salt-sensitive hypertension in maturity, despite the rats resuming a diet that was free of fructose and had normal salt content during young adulthood. The elevation in mean and systolic blood pressures in FCS-GHS and FHS-GHS rats was comparable to rats that had ingested high-fructose and high-salt diet throughout the entire protocol. The cardiovascular parameters such as aortic and renal artery compliance, LV mass and wall thickness, and LV diastolic function were impaired only in the rats that had ingested high fructose and high salt during adolescence. Notably, the magnitude of salt-sensitive blood pressure elevation was similar in all groups fed fructose in early life. Taken together, these findings suggest that the reduced vascular compliance and LV diastolic dysfunction are not simply due to the elevated blood pressure or fructose alone in early life but to the combination of fructose with high salt in adolescence.

An extensive body of work has accrued to show that maternal exposure to environmental and dietary conditions can profoundly influence not only fetal development but also subsequent cardiac and renal function in offspring [31]. Importantly, moderately high maternal salt with [32] or without [33,34] concurrent fructose intake leads to hypertension in male rats. In contrast, the impact of factors on cardiovascular function in the adolescent period of plasticity has received scant attention.

The comparable increases in MAP in each of the four groups during Phase I was consistent with the ~10 mmHg increase typically observed as rats grow and mature [35]. Except for the FHS-FHS group that entered Phase III with elevated blood pressure, rats in the other groups, all of which were on GCS during Phase II, entered Phase III with normal MAPs similar to the GCS-GCS control group. The hypertension that developed in response to high-salt intake in rats that ingested fructose during the critical developmental period was driven by elevation in systolic pressure to levels equivalent to the rats that had consumed fructose and high salt throughout. Slight increases in diastolic blood pressure occurred which prevented any statistically significant increases in pulse pressure which were, nonetheless, nearly two-fold higher than in the GCS-GCS group. Importantly, systolic blood pressure and pulse pressure are strongly correlated with subsequent major adverse cardiac events [36,37]. Thus, fructose alone or combined with high salt during the critical adolescent period predisposes to salt sensitivity and hypertension in maturity.

The mechanisms underlying later salt-sensitive hypertension in rats that consumed fructose in youth remain to be defined. Failure to suppress PRA in the FHS-GHS and FHS-FHS suggests involvement of the renin-angiotensin system. Angiotensin II serves as a pressor inducing hormone that can act systemically on the vasculature to increase vasoconstriction or on target organs such as the kidney to increase extracellular fluid volume by facilitating fluid reabsorption [38]. Notably, adult rats fed similar fructose and high-salt diets exhibit increased proximal tubular sodium-hydrogen exchange [39,40,41,42] and stimulation of thick ascending limb sodium-potassium-2-chloride cotransporter expression [43] as well as enhanced renal sympathetic nerve activity [7]. Increased extracellular volume due to positive net sodium balance together with neurohumorally mediated vasoconstriction over the course of Phase III in rats fed a high-fructose diet in Phase I likely plays a role in producing the increases in MAP [6]. However, increased extracellular volume is unlikely to be the only governing factor. Prolonged fructose feeding has been associated with hyperinsulinemia which can cause increased levels of other vasoactive factors such as endothelin-1 [28], reactive oxygen species and uric acid [4,27,44,45,46,47]. Although no differences in basal plasma glucose or insulin levels were observed among all four groups, the significantly lower glucose:insulin ratio in the groups that consumed fructose and high salt in adolescence indicates a possible role for insulin resistance, a hallmark of pre-diabetes. Whether these or other mechanisms remain “primed” by high-fructose intake during the plastic adolescent period and are then brought into play to induce hypertension upon ingestion of high-salt diet in the absence of fructose later in life will need further investigation.

Increased arterial pressure over time can induce cellular and molecular alterations that deform the vascular wall and increase afterload to the left ventricle [48]. It is noteworthy that the FCS-GHS group displayed hypertension equivalent to that of the other fructose-fed groups by the end of Phase III, but vascular stiffening and LV diastolic dysfunction occurred only in the groups exposed to both fructose and high salt in early life. In fact, evidence of vascular dysfunction became evident in FHS groups by the end of Phase I as demonstrated by increased PWV despite similar arterial pressures across all groups. In Phase III, PWV is augmented in both FHS-GCS and FHS-FHS groups when compared to Phase III GCS-GCS controls. Notably, in rats fed fructose and high salt for the entire protocol, the decline in aortic compliance progressed further in Phase III when compared to initial Phase I measurements. These findings suggest that the combination of fructose and high-salt diet has a direct effect on vascular function that is independent of the elevated arterial pressure. Failure of optimal suppression of PRA in the FHS-GHS and FHS-FHS groups supports an angiotensin-associated mechanism underlying the reduced arterial compliance in these groups, but does not exclude additional potential mechanisms such as increased sympathetic activity [7] or increased sodium reabsorption that could lead to an expanded extracellular volume [39,40,41]. Notably, these mechanisms are not necessarily independent of each other as increased renal sympathetic activity enhances renin secretion and Ang II increases proximal tubule sodium reabsorption by the kidney. Importantly, the present data indicate that fructose alone during the early adolescent phase does not impair the normal suppression of PRA with ingestion of high salt later in life. Rather the combined ingestion of fructose and high salt in this early plastic phase does predispose to salt-sensitive hypertension later in life.

Several studies have demonstrated the prognostic ability of the renal resistive index (RRI) to predict the decline in renal function associated with the progression of hypertension, chronic nephropathy, and diabetes mellitus in humans [49,50,51,52] and adverse cardiac and renal outcomes in hypertension [53,54]. While some controversy remains over the reliability of RRI as a measurement across all diseases [55], there is a general consensus that elevated RRI is linked closely with systemic vascular stiffness. Additional studies have found functional correlation between elevated RRI and intrarenal perfusion as well as histopathological findings such as tubulointerstitial damage and renal atherosclerosis [56,57,58].

Consistent with PWV, we observed significant increases in RRI in each of the groups fed fructose in early adolescence only in Phase III. Importantly, as a measure of pulsatility, RRI reflects intrinsic renal artery compliance but is also influenced substantially by changes in upstream systemic and downstream intrarenal vascular properties [26]. The elevated RRI is thus consistent with the increased aortic PWV observed in this present study but suggests that the decline in renal artery compliance is delayed compared with changes in the ascending aorta and aortic arch where hydrostatic and shear forces are greater [59]. Oxidative stress [27,47], impaired nitric oxide generation [6], and inflammatory mechanisms [60] have been implicated in vascular changes during fructose and high-salt exposure. Again, whether these same factors contribute to the impaired compliance of the aorta and renal artery observed after exposure to fructose and high salt in youth is likely but remains to be proven.

Total peripheral resistance is a function of MAP and heart rate—an increase in either factor without a corresponding decrease in the other elevates total peripheral resistance [61]. In the present study, this physiologic dysfunction was observed as both an increase in systemic resistance and lack of vascular compliance. The net effect of these factors was an increase in ventricular afterload leading to left ventricular remodeling and subsequent hypertrophy evidenced by increased LV mass and total wall thickness. Together with the augmented ratio of ventricular wall thickness to end-diastolic cavity radius, these findings are consistent with concentric remodeling with preserved ejection fraction [62,63]. The decrease in the ratio of early to late diastolic filling was accompanied by an increase in isovolumetric relaxation time and decrease in mitral valve deceleration time. Shortening of the mitral valve deceleration time implies restrictive filling and has been positively correlated to severe adverse cardiac events [64]. Each of these measurements are indicative of diastolic dysfunction and are phenotypes associated with either the development of cardiomyopathy in rats that had consumed fructose and high salt in the critical adolescent period [65,66,67,68]. Despite the lack of rigorous morphometric studies, collagen deposition was apparent only in the two groups of rats fed FHS in early life. The present finding is consistent with the findings of Abdelhaffez et al. [69] who reported increased cardiac interstitial fibrosis after rats ingested 12 weeks of 20% fructose in their drinking water. Unfortunately, that study did not provide functional data. Intriguingly, long-chain non-coding mRNAs that are co-expressed with mRNAs involved the fructose metabolic pathways have been implicated in myocardial fibrosis after myocardial infarction in humans [70]. Whether similar cellular and biochemical pathways are implicated in fructose-high salt-induced cardiovascular dysfunction will be crucial avenues of investigation.

### 4.1. Limitations

The present study used 20% glucose with 0.4% NaCl as the reference group to control for caloric intake rather than a standard rat chow which is typically ~7% simple sugars. Previous studies have found no significant changes in arterial pressure or bodily sodium balance in rats fed 20% glucose with either low- or high-salt diet for short (1 week) or more prolonged periods of time (3 weeks) [6,7]. It should be noted that in these other studies, the sugars (glucose and fructose) were in the drinking water rather than in the chow. Incorporating the carbohydrate in the chow permitted more accurate assessment of intake and equalization across groups. Nonetheless, it is important to note that, while the timeline of the study at present exceeds that of these prior studies, the values for hemodynamic, vascular, and cardiac parameters are comparable in our GCS-GCS rats that we used as reference group to parameters observed after three weeks of 20% glucose in drinking water with either 0.4% or 4% NaCl [27]. The 9- to 10-week exposure to dietary fructose in the FHS-FHS group was certainly longer than that in previous studies that evaluated these cardiovascular parameters. PRA was not suppressed in the FCS-FHS and the FHS-FHS groups. Ideally, concurrent plasma Ang II measurements would have been confirmatory as in our previous studies [7,27]. The volume of plasma required for plasma Ang II measurements by validated assay in our laboratory is 0.8–1.0 mL. Obtaining blood from conscious rats via indwelling catheter while avoiding hypotension that could potentially induce an increase in PRA and Ang II independent of the dietary condition was a primary goal. Due to the need to assess other plasma factors, we were only able to obtain sufficient plasma to reliably assess PRA, which only required 50 µL of plasma. Sex hormones play an important role in the development of hypertension following a high-fructose diet [71,72]. We only studied male rats in the present cohort in part due to restrictions in the number of animals permitted during the pandemic restrictions. We acknowledge that female rats have been shown to be particularly resistant to the development of insulin resistance and, therefore, may prove to be less prone to the consequences of fructose and high-salt diet [73,74,75]. Studies in female rats will be needed in the future. Finally, the nature of the ultrasonographic imaging precluded obtaining all parameters in each of the rats due to anatomical variations or issues with technique. Blood samples obtained at the end of this study via the indwelling arterial catheter were performed in conscious animals to avoid confounders such as hypotension and anesthesia; however, in some cases, this limited the volume of plasma that could be obtained due clotting or kinking of the catheter. Although statistical analyses for missing data were performed by imputation using the mean, the limitation still exists.

### 4.2. Perspectives

Pre-clinical and clinical studies have clearly shown the relationship between frank diabetes mellitus and cardiovascular complications [65,76,77,78,79,80]. Insulin resistance in the pre-diabetic state even without frank hyperglycemia may play an important role in developing cardiovascular abnormalities [81]. Alternatively, the exposure to both fructose *and* high salt early in life in Phase I is an important factor for later development of the vasculopathy and cardiomyopathy.

On the other hand, fructose feeding alone, without the addition of high dietary sodium during the critical developmental period is sufficient to induce as state of salt sensitivity later in life which renders the body susceptible to hypertension. Chronically this can lead to various cardiac and renal co-morbidities such as heart failure and chronic kidney disease [82,83]. Even FCS-GHS groups that became hypertensive only later in life had a reduction in diastolic function, indicated by the E/A ratio, but this was not significant. Contrastingly, the addition of high salt to a moderate fructose diet during pubescent, developmental years had lasting effects on cardiac and renal function evidenced by diastolic dysfunction, ventricular hypertrophy, and failed renin suppression. This detriment occurred in rats that were fed this fructose and high-salt diet chronically and those that were allowed a period of reprise from poor dietary conditions (FHS-GHS) groups. Indeed, diets with fructose fed early in life-with or without the presence of elevated sodium—promote adaptations that render the body increasingly vulnerable to complications caused by even modest dietary insults; these insults are long lasting and with severe consequence.

## 5. Conclusions

In summary, consumption of 20% fructose but not glucose by male rats during pre- and early adolescence, a proportion of caloric intake comparable to the upper quintile of humans, results in salt-sensitive hypertension in mature animals. When ingested together with a high-salt diet during this critical plastic phase, dietary fructose also predisposes to vascular stiffening and left ventricular diastolic dysfunction in later life.

## Figures and Tables

**Table 1:** Initial and Final Body Weights, Heart Weights, Glycemic Parameters and Plasma Renin Activity among the Four Groups of Rats.

Dietary Regimen	*n*	Initial Weight (g)	Final Weight (g)	Heart Weight (g/kg)	Fasting Glucose (mg/dL)	Fasting Insulin (ng/mL)	G:I Ratio (×10^6^)	PRA (ngAngI/mL/hr)
**GCS-GCS**	9	125 ± 4	381 ± 9	3.3 ± 0.1	128 ± 13	1.14 ± 0.23	64.7 ± 6.4	1.82 ± 0.20
**FCS-GHS**	9	132 ± 4	347 ± 10	3.4 ± 0.1	129 ± 24	0.74 ± 0.12	74.6 ± 12.6	0.66 ± 0.12 *
**FHS-GHS**	8	128 ± 3	362 ± 12	3.2 ± 0.1	126 ± 14	1.33 ± 0.12	31.6 ± 1.9 *^,†^	1.35 ± 0.28
**FHS-FHS**	9	127 ± 5	366 ± 12	3.4 ± 0.1	118 ± 11	1.03 ± 0.17	39.1 ± 7.1 ^†^	1.09 ± 0.29

GCS-GCS, 20% glucose + 0.4% NaCl in Phases I–III; FCS-GHS, 20% fructose + 0.4% NaCl in Phase 1 and 20% glucose +4% NaCl in Phase III; FHS-GHS, 20% fructose + 4% NaCl in Phase 1 and 20% glucose + 4% NaCl in Phase III; FHS-FHS, 20% fructose + 4% NaCl in Phases I–III. PRA, plasma renin activity; G:I Ratio, glucose:insulin ratio. All groups except FHS-FHS were on 20% glucose + 0.4% NaCl in Phase II. Due to lack of sufficient plasma for insulin, *n* values for insulin and G:I Ratio are as follows: 5, 6, 7 and 7. Values are the mean ± SE; *n* as indicated per group. * *p* < 0.05 vs. GCS-GCS. ^†^ *p* < 0.05 vs. FCS-GHS.

**Table 2:** Daily Caloric and Sodium Consumption.

				**PHASE I**			
		**WEEK 1**		**WEEK 2**		**WEEK 3**	
**Dietary Regimen**	***n***	**Caloric Intake (kcal/day)**	**Sodium Intake (mmol/day)**	**Caloric Intake (kcal/day)**	**Sodium Intake (mmol/day)**	**Caloric Intake (kcal/day)**	**Sodium Intake (mmol/day)**
**GCS-GCS**	9	61.5 ± 1.7	3.0 ± 0.3	68.4 ± 1.3	3.0 ± 0.1	66.0 ± 2.1	2.9 ± 0.1
**FCS-GHS**	9	60.7 ± 2.6	2.7 ± 0.1	67.2 ± 1.4	3.0 ± 0.1	65.7 ± 2.0	3.2 ± 0.3
**FHS-GHS**	8	48.7 ± 1.6 *^,†^	23.5 ± 0.8 *^,†^	60.6 ± 2.2 *	29.2 ± 1.1 *^,†^	61.8 ± 2.1	29.3 ± 0.9 *^,†^
**FHS-FHS**	9	51.9 ± 2.1 *^,†^	24.9 ± 1.0 *^,†^	63.9 ± 1.2	30.9 ± 0.6 *^,†^	63.5 ± 1.5	31.0 ± 0.8 *^,†^
				**PHASE III**			
	**WEEK 1**			**WEEK 2**		**WEEK 3**	
**Dietary Regimen**	***n***	**Caloric Intake (kcal/day)**	**Sodium Intake** **(mmol/day)**	**Caloric Intake (kcal/day)**	**Sodium Intake** **(mmol/day)**	**Caloric Intake (kcal/day)**	**Sodium Intake** **(mmol/day)**
**GCS-GCS**	9	62.6 ± 1.9	2.8 ± 0.1	63.5 ± 2.1	2.9 ± 0.1	64.3 ± 1.8	2.8 ± 0.1
**FCS-GHS**	9	45.9 ± 1.7 *	22.1 ± 0.8 *	54.4 ± 1.4 *	26.2 ± 0.7 *	57.1 ± 1.3	27.5 ± 0.6 *
**FHS-GHS**	8	50.1 ± 2.5 *	23.3 ± 1.2 *	56.1 ± 2.2	26.0 ± 0.8 *	59.9 ± 2.0	27.7 ± 0.6 *
**FHS-FHS**	9	60.5 ± 1.6 ^†,§^	27.5 ± 0.8 *	63.9 ± 1.9 ^†,§^	29.0 ± 0.3 *	66.4 ± 2.6 ^†^	29.9 ± 1.0 *

Values are the mean ± SE, *n* as indicated per group. Group names as in Table 1. Caloric intake calculated using caloric profiles of 3.98 kcal/g and 3.61 kcal/g for 0.4% and 4% NaCl chow, respectively. * *p* < 0.05 vs. GCS-GCS; ^†^ *p* < 0.05 vs. FCS-GHS; ^§^ *p* < 0.05 vs. FHS-GHS.

**Table 3:** Hemodynamic Parameters in the Four Groups of Rats at Baseline and End of Phase 1 and Phase 3.

(**A**)						
**Dietary** **Regimen**		**MAP (mmHg**)			**HEART RATE (BPM)**	
	***n***	**Baseline**	**End Phase I**		**Baseline**	**End Phase I**
**GCS-GCS**	9	100.0 ± 1.0	108.4 ± 1.6		465 ± 8	391 ± 16
**FCS-GHS**	9	100.7 ± 0.9	108.0 ± 0.9		458 ± 9	395 ± 15
**FHS-GHS**	8	100.6 ± 1.3	111.1 ± 1.3		456 ± 11	381 ± 8
**FHS-FHS**	9	99.4 ± 1.0	110.2 ± 1.4		451 ± 11	391 ± 11
(**B**)						
**Dietary** **Regimen**	***n***	**Δ MAP** **(mmHg)**	**Δ SBP** **(mmHg)**	**Δ DBP** **(mmHg)**	**Δ HR** **(BPM)**	**Δ PP** **(MMHG)**
**GCS-GCS**	7	10 ± 1.0	11 ± 2.2	11 ± 1.2	−92 ± 11	2.4 ± 1.5
**FCS-GHS**	8	15 ± 0.9 *	18 ± 0.9 *	14 ± 1.3	−103 ± 18	4.2 ± 1.6
**FHS-GHS**	8	15 ± 1.4 *	18 ± 1.7 *	13 ± 1.7	−105 ± 6	4.2 ± 2.0
**FHS-FHS**	8	16 ± 2.0 **	19 ± 2.3 **	14 ± 1.9	−89 ± 14	4.6 ± 1.4

Group names as in Table 1. Values are the mean ± SE, n as indicated per group. (**A**) Mean arterial pressure (MAP) and heart rate (HR) at baseline and at the end of Phase I. (**B**) Study-wide changes in hemodynamics calculated as the difference between measurements taken at the end of Phase III and baseline values at the beginning of Phase I. Values are the mean ± SE, n as indicated per group. * *p* < 0.05 vs. GCS-GCS; ** *p* < 0.01 vs. GCS-GCS.

**Table 4:** Echocardiographic Parameters at the End of Phase III.

	Groups			
	GCS-GCS	FCS-GHS	FHS-GHS	FHS-FHS
***n***	9	8	8	9
**LVEF (%)**	74.9 ± 3.4	77.3 ± 4.4	80.3 ± 3.7	78.2 ± 2.3
**LVFS (%)**	46.0 ± 3.4	49.1 ± 4.4	51.9 ± 4.4	48.8 ± 2.4
**LVID_s_ (mm)**	3.7 ± 0.3	3.4 ± 0.4	3.0 ± 0.4	3.5 ± 0.3
**LVID_D_ (mm)**	6.7 ± 0.3	6.5 ± 0.3	6.1 ± 0.4	6.9 ± 0.4
**LVAW_S_ (mm)**	3.2 ± 0.1	3.3 ± 0.2	3.5 ± 0.2	3.6 ± 0.1
**LVAW_D_ (mm)**	1.9 ± 0.03	2.0 ± 0.1	2.5 ± 0.1 *	2.2 ± 0.1 *
**LVPW_S_ (mm)**	3.5 ± 0.1	3.5 ± 0.2	4.0 ± 0.3	4.0 ± 0.2
**LVPW_D_ (mm)**	2.3 ± 0.1	2.4 ± 0.1	3.0 ± 0.3 *	2.7 ± 0.2
**LVTW_S_ (mm)**	6.8 ± 0.2	6.8 ± 0.4	7.4 ± 0.3	7.6 ± 0.4
**LVTW_D_ (mm)**	4.3 ± 0.1	4.4 ± 0.2	5.6 ± 0.4 *	4.9 ± 0.3
**LV Mass (mg)**	1190 ± 73	1060 ± 59	1401 ± 56 *	1373.0 ± 75 *

Group names as in Table 1: LVEF, left ventricular ejection fraction; LVFS, left ventricular fractional shortening; LVID_s_, left ventricular systolic internal diameter; LVID_D_, left ventricular diastolic internal diameter; LVAW, left ventricular anterior wall width; LVPW, left ventricular posterior wall width; LVTW, left ventricular total wall width; LV Mass, left ventricular mass. Values are the mean ± SE. * *p* < 0.05 vs. GCS-GCS.

**Table 5:** Echocardiographic Parameters Associated with Diastolic Function at the End of Phase III.

	Group			
	GCS-GCS	FCS-GHS	FHS-GHS	FHS-FHS
***n***	9	8	8	9
**Peak E (mm/s)**	706 ± 22	630 ± 45	652 ± 41	641 ± 39
**Peak A (mm/s)**	555 ± 27	550 ± 44	576 ± 34	621 ± 32
**E/A**	1.31 ± 0.06	1.16 ± 0.06	1.14 ± 0.06	1.03 ± 0.04 **
**DT (ms)**	39.5 ± 4.0	28.0 ± 2.2 *	31.8 ± 3.0	25.4 ± 2.3 **
**IVRT (ms)**	25.5 ± 1.0	27.7 ± 1.5	27.6 ± 1.1	29.0 ± 0.9
**E’ (mm/s)**	34.1 ± 2.6	32.3 ± 4.6	34.2 ± 4.0	27.4 ± 1.8
**A’ (mm/s)**	45.3 ± 3.7	45.8 ± 4.7	48.0 ± 4.9	47.1 ± 4.7
**E/E’**	22.5 ± 1.9	25.2 ± 6.2	21.1 ± 3.3	24.3 ± 2.3
**E’/A’**	0.81 ± 0.01	0.71 ± 0.09	0.81 ± 0.15	0.64 ± 0.01

Group names as in Table 1: E, early phase ventricular filling; A, late phase ventricular filling; DT, mitral valve deceleration time; IVRT, isovolumetric relaxation time; E’, mitral annulus early phase filling; A’, mitral annulus late phase filling. Values are the mean ± SE. * *p* < 0.05 vs. GCS-GCS, ** *p* < 0.01 vs. GCS-GCS.

## Data Availability

The data presented in this study are available on request from the corresponding author by formal request to the Research and Development Office of the John D. Dingell VA Medical Center, Detroit, Michigan.
